# Nutritional treatment of advanced CKD: twenty consensus statements

**DOI:** 10.1007/s40620-018-0497-z

**Published:** 2018-05-24

**Authors:** Adamasco Cupisti, Giuliano Brunori, Biagio Raffaele Di Iorio, Claudia D’Alessandro, Franca Pasticci, Carmela Cosola, Vincenzo Bellizzi, Piergiorgio Bolasco, Alessandro Capitanini, Anna Laura Fantuzzi, Annalisa Gennari, Giorgina Barbara Piccoli, Giuseppe Quintaliani, Mario Salomone, Massimo Sandrini, Domenico Santoro, Patrizia Babini, Enrico Fiaccadori, Giovanni Gambaro, Giacomo Garibotto, Mariacristina Gregorini, Marcora Mandreoli, Roberto Minutolo, Giovanni Cancarini, Giuseppe Conte, Francesco Locatelli, Loreto Gesualdo

**Affiliations:** 1 Dipartimento di Medicina Clinica e Sperimentale, Università di Pisa, Via Roma 67, 56126 Pisa, Italy; 2 SC Multizonale di Nefrologia e Dialisi, APSS, Trento, Italy; 3 UOC di Nefrologia, PO “A. Landolfi”, Solofra, AV Italy; 4 ANDID Associazione Nazionale Dietisti, Verona, Italy; 5 Dipartimento di Medicina, USL Umbria 1, Perugia, Italy; 6 Dipartimento dell’Emergenza e dei Trapianti di Organi D.E.T.O. - Sezione di Nefrologia, Dialisi e Trapianti, Università degli Studi di Bari Aldo Moro, Bari, Italy; 7 Divisione di Nefrologia, Dialisi e Trapianto Azienda Ospedaliera Universitaria “San Giovanni di Dio e Ruggi d’Aragona” Salerno, Salerno, Italy; 8 Nefrologia e Dialisi, ASL Cagliari Consultant, Cagliari, Italy; 9 SOS Nefrologia Pistoia, ASL Toscana Centro, Florence, Italy; 10 Nutrizione e Dietetica Aziendale, AUSL Modena, Modena, Italy; 11 UO Nefrologia, ASST Spedali Civili e Università di Brescia, Brescia, Italy; 12 Dipartimento di Scienze Cliniche e Biologiche, Università di Torino, Turin, Italy; 13 Centre Hospitalier Le Mans, Le Mans, France; 14 Nefrologia e Dialisi, Azienda Ospedaliera di Perugia, Perugia, Italy; 15 Nefrologia e Dialisi, ASL TO5, Chieri, TO Italy; 16 Dipartimento di Medicina Clinica e Sperimentale-UOC di Nefrologia e Dialisi, Università di Messina, Messina, Italy; 17 ANED Onlus, Milan, Italy; 18 Unità di Fisiopatologia Insufficienza Renale, Università di Parma, Parma, Italy; 19 Dipartimento di Medicina, Università Cattolica del Sacro Cuore, Rome, Italy; 20 Università degli Studi di Genova, DIMI and IRCCS AOU San Martino IST, Genoa, Italy; 21 SC Nefrologia e Dialisi Arcispedale S. Maria Nuova Azienda USL Reggio Emilia, Reggio Emilia, Italy; 22 Nefrologia e Dialisi, Ospedale S. Maria Scaletta, Azienda USL Imola, Imola, Italy; 23 Divisione di Nefrologia, Università della Campania “Luigi Vanvitelli”, Naples, Italy; 24 SC Nefrologia e Dialisi, Ospedale Manzoni, ASST, Lecco, Italy

**Keywords:** CKD, Nutritional treatment, Diet, Dialysis, Kidney transplant, Chronic renal failure

## Abstract

The Italian nephrology has a long tradition and experience in the field of dietetic-nutritional therapy (DNT), which is an important component in the conservative management of the patient suffering from a chronic kidney disease, which precedes and integrates the pharmacological therapies. The objectives of DNT include the maintenance of an optimal nutritional status, the prevention and/or correction of signs, symptoms and complications of chronic renal failure and, possibly, the delay in starting of dialysis. The DNT includes modulation of protein intake, adequacy of caloric intake, control of sodium and potassium intake, and reduction of phosphorus intake. For all dietary-nutritional therapies, and in particular those aimed at the patient with chronic renal failure, the problem of patient adherence to the dietetic-nutritional scheme is a key element for the success and safety of the DNT and it can be favored by an interdisciplinary and multi-professional approach of information, education, dietary prescription and follow-up. This consensus document, which defines twenty essential points of the nutritional approach to patients with advanced chronic renal failure, has been written, discussed and shared by the Italian nephrologists together with representatives of dietitians (ANDID) and patients (ANED).

## Introduction

Dietetic-nutritional therapy (DNT) is an important component of conservative treatment of patients suffering from chronic kidney disease (CKD) which must anticipate and be integrated with pharmacological therapies. Objectives of DNT are to maintain an optimal nutritional state, prevent and/or correct the signs, symptoms, and complications related to chronic renal insufficiency, and delay the start of dialysis. Furthermore, DNT enables a reduction in the drug load (and related side-effects and interactions) and can allow for safe and effective use of lower doses of dialysis, even when the glomerular filtration rate (GFR) continues to diminish. Conservative treatment methods and incremental dialysis can improve quality of life and reduce health care costs. Recently, it has also been reported that proper nutrition and lifestyle, such as the Dietary Approaches to Stop Hypertension (DASH) diet and our own “Mediterranean Diet”, are able to reduce the incidence of CKD as well as cardiovascular risk.

Italian nephrologists have a long tradition of and experience with DNT, founded on, but not limited to, reduction of protein intake. In fact, the concept of DNT also includes an adequate calorie intake, control of sodium and potassium intakes, and reduction of phosphate intake. Besides the quantitative aspects, dietary support also calls for modification in the quality of food, in favor of foods of plant origin which induce favorable effects on phosphate metabolism and acid-base balance with a better control of arterial pressure and renal hemodynamics.

For all nutritional therapies, especially those for patients with CKD, the patient’s adherence to the dietary plan of the DNT is a fundamental element for its success and safety. Implementation of an interdisciplinary and multi-professional approach to information, education, dietary prescription, and follow-up represents a key element for better diffusion and success of DNT in the nephrological field.

This consensus paper is an example of the difficulty of demonstrating what is obvious in the clinical practice and the great need for further study in order to improve our understanding and knowledge. The Italian Society of Nephrology (SIN), through the Working Group on Conservative Therapies of Chronic Kidney Disease, sought to define several aspects of the nutritional approach in patients with advanced chronic renal insufficiency. A consensus statement consisting of 20 essential points was composed, discussed, and shared with dietitians and patients by the National (Italian) Association of Dieticians (ANDID) and the National (Italian) Association of Hemodialysis, Dialysis, and Transplant (ANED). This is the first document jointly shared by the nephrological scientific community, dieticians, and patients concerning essential aspects of the nutritional approach during the advanced stages of CKD.

## In patients with CKD 4–5, a diet with non-monitored intake of calories, protein, sodium, and phosphates hastens and exacerbates clinical and metabolic alterations related to advanced CKD

As CKD progresses, especially in its most advanced stages, various kidney functions tend to become progressively more inefficient. In fact, a progressive incapacity to eliminate elevated amounts of sodium, water, potassium, phosphorus, and hydrogen ions [1] with a tendency towards retention is observed. Uncontrolled intake of nutrients and proteins favors the onset of metabolic and clinical alterations of the uremic status. More specifically, an excess of sodium and water is responsible for the onset of arterial hypertension, edema and heart failure as well as an increase in oxidative stress [2]. A positive balance of phosphorus causes secondary hyperparathyroidism and calcification of the arteries and heart valves, with an increase in cardiovascular mortality [3]. Reduced capacity to eliminate the fixed acid load, derived from protein catabolism, leads to an accumulation of acids with consequent metabolic acidosis [4]. Metabolic acidosis stimulates protein and muscular catabolism, bone demineralisation, insulin resistance, hyperkalemia, etc. [5]. Elimination of nitrogenous waste products from catabolism of proteins and amino acids also diminishes, resulting in its retention together with that of “uremic toxins”, including urea, indole compounds, cresols and guanidines [6]. Accumulation of these substances contributes to the onset of anorexia, nausea, and vomiting, with a subsequent reduction in intake of energy, proteins, and other nutrients [7]. Together, these factors lead to a progressive reduction of protein and energy body stores, leading to protein-energy wasting (PEW) and cachexia, which result in higher rates of hospitalization and mortality [8]. They also contribute to protein-energy depletion, progressive reduction of physical activity and onset of a micro-inflammatory state, more frequent in the advanced stages [9, 10].

## In patients with CKD 4–5, a diet with non-monitored intake of energy, protein, sodium and phosphate can reduce the effectiveness of drug therapy or require an increase of dose

Excessive energy intake can contribute to obesity and dyslipidemia and exacerbates insulin resistance; it limits the effectiveness of antidiabetic and lipid-lowering treatments and requires a dosage increase. An elevated sodium intake reduces the effectiveness of antihypertensive and antiproteinuric therapies, especially renin–angiotensin–aldosterone system (RAAS) inhibitors, with an increased risk of CKD progression and medications use, specifically diuretics [11–14].

Elevated consumption of phosphorus inhibits the effectiveness of intestinal phosphate binders and/or requires a dosage increase. This contributes to poor control of secondary hyperparathyroidism, reducing the therapeutic safety of use of active vitamin D preparations. A poor control of phosphatemia and parathormone (PTH) is associated with a reduced therapeutic response to erythropoiesis-stimulating agents (ESA) and angiotensin-converting enzyme (ACE)-inhibitors [15–17].

A high intake of fixed acids, associated with animal protein consumption, makes it difficult to prevent metabolic acidosis and requires the use of larger amounts of sodium bicarbonate for its correction [18, 19].

## Lack of metabolic compensation, with the appearance of uremic signs and symptoms, is an indication to begin dialysis treatment in the same way as, and independently of, the level of residual renal function

Dialysis is also initiated at relatively high values of GFR in the presence of uremic symptoms or because of the belief that dialysis provides clinical benefits, better quality of life, and less morbidity and mortality [20]. The relationship between the GFR level at the start of dialysis and clinical benefits, however, is controversial. The IDEAL randomized trial evaluated survival rates associated with an early (10–14 ml/min) or late (5–7 ml/min) start and did not demonstrate any advantages or disadvantages associated with the GFR level at the start of dialysis. Initiating dialysis at a lower GFR level does not carry greater risk to the patient [21]. Dialysis has a major impact on the patient’s functional state, especially in very old patients. In fact, after just 1 year, only one out of eight older patients maintained his functional capacity [22]. Dialysis, while improving uremia as well as many other symptoms, is not able to assure an acceptable quality of life in many patients.

Optimization of the conservative treatment for CKD is a rational alternative to early initiation of dialysis. Dialysis should only be started if the uremic symptoms are no longer manageable, independently of the level of renal function [23]. In fact, guidelines indicate clinical cases with certain characteristics (uremic signs and symptoms and possible comorbidities) within a wide range of GFR (6–12 ml/min) who require the start of replacement therapy. Clinical features of untreated uremia (anorexia, hyperazotemia, hyperphosphatemia, metabolic acidosis, hydrosaline retention, malnutrition, etc.) can be controlled with DNT [24].

DNT can postpone, therefore, the start of dialysis even with a reduced GFR, thanks to a better control of the uremic signs and symptoms. When correctly carried out with an adequate energy intake, it does not have negative effects upon the nutritional state and survival rate, both during the CKD phase and after the start of dialysis [25, 26]. The good metabolic control attained with nutritional treatment, enables postponement of the start of renal replacement therapy to a more advanced stage of the illness with no risk to the patient [27, 28].

## Untreated chronic renal insufficiency leads to undernourishment due to loss of appetite, nausea, and anorexia

The natural progression of chronic renal insufficiency leads the patient to reduce intake of calories and protein with the progressive reduction of residual renal function [29, 30]. Alterations caused by renal insufficiency compromise appetite and nutritional status, leading to cachexia and malnutrition [31]. Loss of appetite, anorexia, nausea, or vomiting can be caused by uremic toxicity and by decompensated metabolic acidosis, both indicators for the start of dialysis. In the past, anorexia associated with CKD was attributed to the retention of “medium sized molecules” [22, 32, 33]. More recently, experimental models of chronic uremia suggest that alterations to the complex neuroendocrine pathways operating mainly at the hypothalamic level, could be the basis for anorexia.

In fact, substances which accumulate over the course of advanced CKD, such as hormones (insulin, leptin, PYY3-36 produced by the colon, ghrelin) and uremic toxins (cresols, indoles, and phenols) could be responsible for the anorexia through neuroendocrine mechanisms [34] involving the melanocortin 4 receptor (MC4-R). Increased stimulation of this receptor, suppressing activity of AMP-activated protein kinase (AMPK), determines a reduction in food consumption.

Other conditions which cause anorexia include delayed gastric emptying (as in diabetic gastroparesis), changes in taste, uremic halitosis, uremic gastritis, and the high number of tablets consumed by patients. Chronic inflammation, comorbidity, and depression, as well as difficult socio-economic situations, can also contribute to malnutrition.

An adequate dietetic-nutritional intervention, with indication of the correct quantity and quality of protein intake coupled with an adequate energy intake and the possible addition of sodium bicarbonate supplements (improving metabolic acidosis and reducing uremic intoxication), is a therapeutic tool able to reduce anorexia and protein-energy depletion in patients with advanced chronic renal insufficiency [35].

## Considering the physiopathology of advanced chronic renal insufficiency, an adequate dietetic-nutritional therapy provides: reduction of protein intake, reduction of phosphorus intake, reduction/monitoring of sodium intake, monitoring of potassium intake, and limitation of the fixed acid load

DNT in advanced CKD must be based on limitations of protein intake, especially of animal origin, phosphorus, and sodium, control of potassium, and satisfaction of energy requirement. Such an approach has a precise rationale in human physiology: if renal function diminishes, consequently the filtered load must be reduced in order to provide residual nephrons with adequate control of the excretion of uremic toxins and fixed acids [24]. Appropriate management of DNT for CKD in advanced stages requires a reduction in protein intake to below 0.8 g/kg/die, which corresponds to the recommended intake for the healthy general population [36]. There is no scientific rationale for a high-protein diet even in cases of elevated proteinuria [24]. Rather, evidence exists which indicates that a reduction in food proteins could have antiproteinuric effects [17, 37].

Control of phosphorus intake should begin during the initial stages of CKD. In fact, under normal physiological conditions, a healthy kidney is able to regulate urinary secretions of phosphates with food intake, but the progressive loss of glomerular filtrate, due to CKD progression, rationalizes a reduction of phosphorus intake to below 700 mg/die, the recommended level for the general adult population [36]. Surely, it is useful to educate patients to avoid consuming “hidden” phosphates through additives found in preserved foods or soft drinks [38, 39]. Choosing foods with a lower phosphorus content and using foods of plant origin is useful in limiting the net phosphorus load [40]. Finally, food preparation recommendations are also important [41]; it has been well documented that boiling food results in demineralization.

Regulating sodium intake is essential for a population in which hypertension is almost always present. Limiting sodium consumption can improve the protective effects of RAAS inhibition and boost antiproteinuric action [42]. Patients with hypertension and CKD should immediately limit sodium chloride food intake to 5–6 g/die (corresponding to a urine sodium excretion of 90–100 mmol/die over 24 h) [43, 44]. A low salt diet should be avoided under all conditions of sodium-dispersing nephropathy to avoid sodium depletion, hypotension, and a worsening in renal function. Furthermore, the combined reduction of sodium chloride and phosphorus food intake can have an antiproteinuric synergistic effect in patients begin treated with ACE-inhibitors or sartans [17].

In advanced stages (CKD 4–5) potassium intake should be modulated based on serum levels and must be reduced if hyperkalemia is > 5.5 mmol/l. In such cases, the possibility of withdrawal or reduction of drug regimens which cause hyperpotassemia (e.g. ACE inhibitors, sartans, anti-aldosterones) should be considered—after correcting the metabolic acidosis—or use of potassium-chelating resins.

A reduced acid load (mainly derivatives of proteins from animal-based food sources) is essential in order to reduce accumulation and therefore to prevent or correct metabolic acidosis [19]. A diet rich in vegetables is the most natural way to provide stability without resorting to drugs [18]. Reduction in the net acid load obtained with a vegetarian diet resulted in a 50% reduction in bicarbonate prescriptions [19] and improvement in insulin resistance in diabetics [45]. Another important fact is that correction of metabolic acidosis (obtained with administration of sodium bicarbonate or with fruits and vegetables) can reduce the loss of GFR in subjects with CKD [18]. For this reason, oral bicarbonate integration and consuming fruits and vegetables is important, while at the same time paying close attention to potassium serum levels. Risk of hyperpotassemia is mostly associated with taking anti-aldosterones and/or ACE inhibitors and sartans.

## To ensure the effectiveness of dietetic-nutritional therapy in chronic renal insufficiency, compliance with the following conditions must be verified: satisfaction of the caloric needs, adequate intake of essential amino acids, correction of metabolic acidosis, good glyco-metabolic control

Equilibrium of the nitrogen balance is an essential element in maintaining a good nutritional status and body composition. In a stable renal patient, regardless of acute conditions such as fever, sepsis, burns, surgical interventions, or steroid therapy, even with a reduced protein intake, the nitrogen balance is maintained thanks to a metabolic adaptation mechanism, consisting in the organism’s capacity to reduce protein catabolism [46–49]. This adaptation to the low-protein diet is hindered by conditions which increase the nitrogen demand, such as those analyzed below.

An energy intake lower than  the organism’s needs results in the catabolism of protein for energy production: the greater the energy deficit, the greater the amount of protein used. An elevated energy  intake spares protein and allows for a safe reduction in its intake [50]. An inadequate exogenous supply of essential amino acids increases endogenous nitrogen catabolism. Although reduced in quantity, protein intake must guarantee the needs of essential amino acids in the form of natural foods or pharmacological supplementation.

Metabolic acidosis accelerates protein and muscle amino acid catabolism, stimulating the ubiquitin–proteasome pathway, which prevents adaptation to the hypoproteic diet. Correction of metabolic acidosis is therefore an essential prerequisite for nutritional safety of a normo/low protein regime [51–53].

Insulin is an anabolic hormone that stimulates the uptake of amino acids, increases synthesis and reduces protein catabolism: as a result, protein intake requirements increase in order to maintain the nitrogenous balance in the presence of insulin resistance or poor glycometabolic control in diabetic patients [54].

## Protein-free products, made up of carbohydrates, contain almost no protein, phosphorus, sodium, and potassium. They raise caloric intake while leaving more space for foods rich in high biological value proteins which guarantee essential amino acid intake. Better therapeutic efficacy is thus obtained with a lower risk of nutritional inadequacy

Low protein foods are fundamental to safeguard the correct elaboration and implementation of a low protein diet in patients with chronic renal failure. The use of protein-free products allows for an adequate energy intake by excluding/reducing cereals and derivatives that contain low biological value proteins while maintaining consumption of high biological value proteins from animal sources. Low protein foods are therefore a source of “clean” energy, free from nitrogenous waste products and with negligible potassium, sodium and phosphorus content. The main obstacles to the widespread use of these products include lack of palatability and consistency [55, 56], high cost and interregional inhomogeneity of delivery methods. Recently, the Italian Ministry of Health modified the national standards of care to include CKD on the list of chronic diseases exempt from copayments. Therefore, a standardization of supply methods throughout Italian national territory is expected [57].

The organoleptic differences between low protein foods and their common counterparts mainly stem from the absence of gluten, a protein that, despite its low biological value, has extraordinary technological properties which are the basis of pasta production and cooking processes as well as bread making processes [58, 59]. The absence of gluten limits the consistency, aroma, fragrance and appearance of these products. In recent years, however, the food industry has developed alternative production methods, implementing new production technologies and adding substitute ingredients such as fibers, with good results [55, 60, 61].

## Essential amino acid and ketoacid mixture is an useful supplementation in CKD patients; it is mandatory on a very low-protein diet

Among dietary regimens proposed to patients with CKD 4–5, an important role is played by nutritional therapies with a reduced protein intake (0.3–0.6 g of protein/kg of body weight) and integrated with use of essential amino acids (EAA) and ketoacids (KA) mixtures [24, 41]. These nutritional treatments are to be used in motivated patients, who have good compliance and do not present severe comorbidities [26, 62]. By convention, the standard low protein diet is based on a contribution of 0.6 g of protein/kg of body weight; a very low protein diet (VLPD) is characterized by a contribution of less than 0.3 g of plant-based protein/kg of body weight [63]. A very low protein and vegetarian diet supplies an inadequate amount  of amino acids and so the supplementation with KA and EAA is mandatory  [64, 65]. The use of these mixtures can also be useful in all cases of insufficient spontaneous intake of essential amino acids. In the supplemented very low protein diet (sVLPD), one KA and EAA tablet is prescribed for every 5 kg of ideal body weight. One tablet of KA and EAA contains 500 mg of mixture and provides an intake of 45 mg of calcium salts [66].

Finally, KA and EAA mixtures have a pharmacological effect as they contain a significant amount of keto-leucine, which has an inhibitory action on protein degradation, while leucine is able to promote protein synthesis at the muscle level [67].

Therefore, the amino and keto-acid tablets used in nutritional therapies, with low or very low protein intake, satisfy EAA needs, allowing for maximum limitation of nitrogen, phosphorus and fixed acids load [68, 69].

## DNT in CKD 4–5 must be managed according to the stages and criteria of any other drug therapy: indications, contraindications, side effects, changes in dosage, verification of results, follow-up

Indications for DNT include the existence of metabolic and hydroelectrolytic alterations or metabolic acidosis, protein-energy depletion or obesity, signs and symptoms of uremic intoxication, and the desire or necessity to delay the start of replacement therapy (dialysis or transplant) [24, 28, 70]. Contraindications include the patient’s refusal or inability to follow dietary rules due to socio-economic or psychological distress, a chewing disorder, lack of motivation, deterioration in the quality of life, etc. Side effects which may limit the duration of and adherence to the nutritional treatment include weight loss due to reduced energy intake, caused by poor palatability and loss of appetite, dietetic monotony or difficulty in implementation, depression, and a relationship problems [56, 71]. If these issues are not resolved, they can and should lead to a revision and/or suspension of DNT.

Changes in dosage: changes in intake of protein, calories, or other nutrients such as phosphorus, sodium and potassium must be adjusted according to the clinical needs of each patient and should not be determined a priori by the level of RRF [72, 73]. Verification of results: this is done using indicators such as urea, phosphorus, PTH, hemoglobin, bicarbonate, albumin, body weight, blood pressure, need for dialysis, quality of life, etc. [74]. Follow-up: scheduling of clinical, biochemical and nutritional check-ups, based on the level of residual renal function (RRF), type of DNT and clinical profile. Interactive educational programs should be conducted engaging various professional figures involved in clinical management of CKD, in order to improve knowledge, self-management, and results of conservative therapy in patients with chronic renal failure. Direct patient involvement in diagnostic and therapeutic decision-making processes is also crucial  [75, 76].

## Regular evaluation of the nutritional and functional status of patients with CKD 4–5 at the start of treatment and during follow up is essential for diet management

In the advanced stages of CKD,  PEW is a quite common finding and it represents a negative prognostic factor. It is important to predict, diagnose, and characterize PEW and monitor the response to nutritional therapy. For assessment of nutritional status, several parameters that fall into four broad categories, must be used: (1) body mass; (2) muscle mass; (3) biochemical data; and (4) dietary intakes. According to a recent report [8], PEW can be diagnosed if 3 signs/symptoms from the various categories are detected 3 times over 2–4 consecutive weeks.

Body mass: body weight and its variations and body mass index (BMI) fall into this category. Weight is the simplest and most effective indicator of an adequate calorie intake and should be measured by the patient at home each day and at each visit. Both weight and BMI are influenced by hydration status and do not provide information about body composition. Muscle mass and fat mass: these can be estimated by measuring body circumferences and skinfold thickness. Limits of body fat calipers include the shape of the tool and the presence of significant or anasarcatic edemas [77]. Bio-impedance analysis can be used to assess body composition: it is an easy and repeatable clinical method, but it estimates body composition based on mathematical algorithms which limits its precision and reliability [78]. Biochemical data: this includes monitoring of albumin, prealbumin, cholesterol and transferrin. Conditions such as inflammation, level of renal function, or the “iron asset” limit, however, sensibility and specificity. Dietary intakes: these are measured through diet history, 24-h recall and food diaries [79]. Measurement of urea, sodium, and phosphorus from 24-h urine collection is useful for the objective evaluation of diet intakes [80]. Additional methods for assessing nutritional status include the Subjective Global Assessment (SGA) [81] and the Malnutrition Inflammation Score (MIS) [82].

Recently, functional and performance evaluations have also been recommended. Proposed tests include the Barthel Index, the Karnowsky Scale, the handgrip test, the Sit-To-Stand Test, the 6-min Walking Test, the Rapid Assessment of Physical Activity (RAPA), pedometers, etc. [83]. Regular assessment of a patient’s nutritional and functional status allows for prompt intervention and changes to the prescribed diet to delay the start of dialysis. A renal dietitian should work with the patient’s nephrologist in order to actualize the safest and most effective nutritional interventions in patients with CKD [84].

## Appropriate low protein nutritional therapy does not cause malnutrition in the short or long term

In  stable CKD subjects, the minimum amount of dietary protein needed to maintain a nitrogen neutral balance is about 0.55 g/kg body weight [50]. Protein metabolism is closely linked with energy intake and the nitrogen demand inversely correlates with the energy  supply. Most patients with advanced CKD can maintain a neutral or positive nitrogen balance with 0.55 g/kg/day of protein only if they have a calorie intake of above 30 kcal/kg/day [50]. With a hypoproteic diet, if the patient does not meet daily caloric needs, the nitrogen balance becomes negative with protein degradation and loss of lean body mass.

Most patients with CKD who are prescribed a low-protein  diet have a higher protein intake than prescribed, while energy  intake is very often reduced below the safety threshold [85, 86]. Inability to maintain a sufficient caloric intake is due to factors largely related to uremic toxicity (e.g. anorexia, nausea, asthenia, depression, abnormalities of taste and smell).

Strict adherence to the calorie prescription (35 kcal/kg/day in subjects aged < 60 years and 30 kcal/kg/day in subjects > 60 years) is essential to maintain neutral or positive nitrogen balance during a low-protein  diet; prescription of a personalized nutritional therapy, with counseling and close nutritional monitoring by expert dieticians, allows for prompt identification of anomalies and food errors and thus reduces risk of malnutrition [24].

In a randomized study involving more than 400 patients with CKD stage 3–5 who were monitored for more than 30 months, only 3 subjects developed malnutrition [87], even with very low protein diets, both during the pre-dialysis phase and after beginning dialysis [88, 89]. Furthermore, a very low-protein  diet prolonged up to the start of dialysis does not increase the risk of death during the subsequent period of dialysis [26]. In contrast, about half of patients with advanced CKD left on a free diet spontaneously reduce protein and energy intake, and therefore have a high risk of malnutrition [30, 86]. Properly prescribed DNT with careful clinical monitoring prevents malnutrition and is safe in both the short and long term [90].

## Proper dietetic-nutritional therapy in advanced CKD may delay the need for replacement therapy. For this reason, its use is especially indicated for patients in the pre-emptive kidney transplant programme

Nutritional status is one of the main determinants, together with cardiovascular comorbidity, of the high mortality of dialysis patients. It has been proven that results of renal transplant are inversely proportional to the length of time on dialysis prior to transplant (the so-called “dialysis vintage”) and are better in those with preemptive transplant [91–94].

Nutritional status also influences renal transplant results, but there is less data and it is less exhaustive [95–97]. In this context, there are few published studies that combine hypoproteic diets, nutritional treatment, and preemptive transplant. The only exception is a 2004 study of 9 diabetic patients awaiting kidney and pancreas transplants, 6 of whom followed a vegan diet supplemented with EAA and KA during the bridge period while awaiting combined transplant [98]. Despite the absence of objective data, the general observations lead to attempts to delay the start of dialysis using an integrated nutritional approach in patients awaiting renal or combined transplant.

The approach followed by the Italian school consists, in general, of a normocaloric diet, reduced in protein and phosphorus, associated with acidosis control and controlled sodium intake, carried out under strict clinical surveillance. Italian experiments, in line with different experiments gathered worldwide, demonstrate the potential for a standard hypoproteic diet, and, in selected cases, a very low protein diet, supplemented with EAA and KA, in different populations of patients with advanced chronic renal disease. This approach does not result in increased mortality after the start of dialysis [26, 99].

Two elements especially favor the systematic use of a regimen adapted to stabilize renal function while awaiting preemptive transplant: the first is the advantage of avoiding dialysis (it must be emphasized that data favorable to preemptive transplant were collected from “standard” 3-times a week dialysis patients); the second consists of therapeutic compliance which is superior in motivated patients with a “positive” outlook. In this regard, it can be expected that patients during the pretransplant phase will closely follow the diet plan, in line with different experiences such as waiting time to maturation of a vascular access or pregnancy [62, 100]. It may be appropriate, therefore to offer DNT to patients who may benefit from a predialytic transplant. A lack of data, however, makes it necessary to invest in studies in order to better define the effectiveness of the nutritional approach in preemptive transplant.

## In selected patients, proper dietetic-nutritional therapy can allow an integrated care approach with an once-a-week  hemodialysis schedule

In the ‘80s and ‘90s, Giovannetti and Locatelli began the Integrated Dialysis Dietary Program (IDDP) in patients with GFR < 3 ml/min/1.73 m^2^, consisting of a weekly hemodialysis session integrated with a very low protein diet equal to 0.3–0.4 g/kg/day (VLPD) supplemented by essential amino acids and their ketoanalogues [101, 102]. Even then, the perception that DNT was an important therapeutic tool for delaying the start of dialysis was established [103–105]. Numerous other reports demonstrate that duration and/or frequency of dialysis produces pro-inflammatory and pro-oxidative cytokine stress which leads a reduction in RRF [106]. In 1998, Locatelli et al. suspended IDDP due malnutrition risk and poor patient compliance, reporting a drop-out rate of 66.6% [107]. A similar approach, called the Combined Diet Dialysis Program (CDDP) [108], has treated more than 156 patients until today. The CDDP modified some aspects of the IDDP: protein intake of 0.6 g/kg/day with free diet on dialysis day, glomerular filtrate between 5–8 ml/min/1.73 m^2^, well above previous experiments. Estimation  of dietary compliance was carried out using the Nitrogen Appearance Area formula and RRF with, on average, three weekly urine collections, using the mean clearance of urea and creatinine. In a controlled, non-randomized study, significant advantages of CDDP were shown when compared with patients on hemodialysis three times a week, based on measurements of β2-microglobulin and phosphatemia and a better preservation of residual diuresis [108]. At 24 months, the cumulative survival among the 38 patients on CDDP and 30 in standard thrice-a-week hemodialysis schedule  was identical. Subsequently, at 96 months of follow-up, the remaining patients showed a higher cumulative survival rate in the CDDP (p < 0.05), highlighting a significant saving on indirect costs (hospitalizations) and reduction of direct costs by 75% [109, 110]. Thus, although the data derives from a non-randomized study, the CDDP in selected, cooperative patients may be the best “bridge” option to starting an incremental hemodialysis program.

## Proper Dietetic Nutritional Therapy can allow an integrated incremental peritoneal dialysis regime

Use of incremental peritoneal dialysis (IPD) is justified by current guidelines that establish minimum purifying targets, by adding peritoneal purification and RRF, that must be reached in order to start with a reduced dialysis dose [111]. IPD has not yet been defined conclusively. The prevailing definition is 1–2 daily treatments for continuous ambulatory peritoneal dialysis (PD) and a maximum of 4 weekly sessions in automated PD. It must be emphasized that IPD is not early dialysis and requires monthly monitoring of RRF to promptly adjust dialytic dosage. Despite little evidence in publications, IPD is widely used, mostly in Italy [112]. IPD has resulted safe for patients and has shown advantages in terms of lower hospitalization, incidence of peritonitis and rate of decrease in RRF [113], as well as a lower impact on patient quality of life.

Indications for DNT are well defined for CKD 5 [43] and for standard PD [114]. Experiences of association between DNT and incremental hemodialysis are available [109]. With regard to IPD, there are no specific indications to date. DNT in IPD could reduce possible occurrence of uremic symptoms, improve metabolic control, and contribute to a delay in RRF decline. The main problems could be poor tolerance or adherence to DNT or risk of worsening nutritional status and it therefore would require periodic metabolic and anthropometric checkups [28]. In Brescia, an IPD experiment began in 2002. To date, more than 50% of patients start with IPD, prevalently with manual methods. In all cases DNT is initiated or continued with a daily intake of 0.6–0.8 g protein/kg body weight, 30–35 kcal/kg body weight and 5 g/day of NaCl.

Beyond this experience, controlled studies are necessary in order to confirm that correct DNT, associated with close monitoring of nutritional status and RRF, allows for optimization of a IPD regime. After all, the importance of renal clearance as compared to peritoneal clearance is well known.

## Advanced Chronic Kidney Disease is characterized by a dysbiosis of the intestinal microbiota, which contributes to uremic intoxication and cardiovascular damage. A hypoproteic nutritional therapy, associated with adequate fiber intake, can counteract dysbiosis and reduce the production of uremic toxins

In advanced stages of CKD, a state of dysbiosis of the intestinal microbiota occurs, with alteration of intestinal permeability and bacterial composition, imbalance of microbial metabolism in the proteolytic sense, and increased production of uremic toxins, such as p-cresol and indoxyl sulfate [115]. These toxins, normally excreted by the kidney, accumulate in the patient depending on the disease stage and contribute to an accelerated progression towards renal death and inflammatory and cardiovascular complications [116].

Dysbiosis worsened in cases of dietary restriction of plants and fibers in an attempt to control potassium levels [117]. Therefore, the ideal DNT for a patient with advanced CKD should include protein restriction and an intake of 20–30 g/day of dietary fiber, directing the food choices towards those containing less phosphorus and potassium but with the same fiber content [117]. Desirable beneficial effects of this DNT in the advanced CKD could include:^1^

- reduction of intestinal dysbiosis (see footnote 1) [118, 119];
- reduction of circulating uremic toxins [118–121];
- reduction of serum creatinine and BUN [122–124];
- increase in saccharolytic fermentation and short-chain fatty acids (SCFA) at the ascending colon level (see footnote 1) [119];
- increase in intestinal transit [125] and fecal mass with increased excretion of nitrogen compounds [122];
- reduction of inflammation [126];
- potential reduction of intestinal permeability [127];
- potential slowing of CKD progression.

## In terms of pharmacoeconomics, appropriate Dietetic Nutritional Therapy enables cost and resource savings in the management of patients with advanced chronic renal failure

CKD is a social issue (it affects 7–8% of the population) [128], which must be identified early and treated appropriately, preventing its progression towards dialysis in order to reduce morbidity, mortality, and costs.

Screening of the general population is not considered cost-effective [129]. Patients with progressive renal failure who start renal replacement therapy within 4 months of their first nephrologist’s appointment show an increase in costs when compared to early referrals [130]. Dialysis has very high costs compared to predialysis [131, 132]. Data from CENSIS 2008 [133] show that dialysis patient care costs around € 50,000/year (€ 35,000 for peritoneal dialysis), including social care.

Data from the Italian Dialysis and Transplant Registry reports an incidence of about 160 patients per million population (pmp) with an estimate of about 9600 patients who enter dialysis each year and with 40,000 patients on dialysis. If the expenditure is about € 50,000 per patient/year, we can estimate that the total expenditure for dialysis in Italy reaches approximately € 2,000,000,000 per year.

Delaying the start of dialysis by just 1-year results in considerable savings: many patients, especially the elderly who have a high probability of death within the first year of dialysis, could reach end-of-life without ever being subjected to dialysis, avoiding suffering for patients and their families and contributing to the sustainability of the Italian National Healthcare System.

Kidney transplant is the therapy of choice for chronic renal failure and the most economical treatment in the long run. Costs, over a 3-year observation period, amount to € 95,247, € 52,543 of which are related to the transplant itself, specifically surgical procedure and transplant center stay [134].

For all these reasons, delaying the start of dialysis could result in significant economic savings. A Cochrane Meta-Analysis showed that the reduction of protein intake reduces the risk of initiating dialysis by 31%, with a Numbers Needed to Treat (NNT) value of 17. Use of DNT, in a cost-effectiveness analysis, has shown considerable savings [135] considering the quality-adjusted life-years (QALYs) earned in succession. An effective conservative treatment able to postpone dialysis reduces costs up to about € 21,180 per patient in the first year, € 6500 in the second year and € 682 in the third year of treatment, with significant benefits that favor a diet supplemented with ketoanalogues even in worst-case scenarios [136].

A VLPD allows for savings of about € 20,000/year in resource consumption for erythropoietin (EPO) [137]. Also to bear in mind is the fact that the cost of nutritional intervention, which improves quality of care, means a reduction in hospitalization costs before start of dialysis and a timely preparation of vascular access with a savings of venous catheters.

## For a more effective and easier clinical management of advanced CKD, it is necessary to implement organizational models integrating various health professionals

CKD represents a public health problem due to its high prevalence and high impact on the population’s morbidity and mortality [138, 139]. It necessitates development of interventions aimed at early diagnosis, delay of damage progression, and prevention of complications. A nephrologist must coordinate these activities with the aid of other professionals (nurses, psychologists, dieticians, etc.). The most effective tool for this purpose is the creation of integrated (diagnostic, therapy, assistance) care pathways (ICP) in agreement with regional Health Departments.

Pathways of this nature have been initiated in some regions: the Prevention of Progressive Renal Failure (PIRP) project in the Emilia Romagna Region [140] has been active since 2004. Specific clinics have been organized in the region’s nephrological centers and a collaboration with territorial healthcare has been established; the data collected represent an important patrimony for analysis of renal disease progression and the incidence of ESRD has been reduced. The Lombardian Nephrological Network [141] has developed programs to involve general practitioners. Similar experiments are present in other areas of the country. In Piedmont, the Nephrology Network has structured an intervention model with regards to advanced kidney disease, implemented by a Council Decree [142]. This project has contributed to the creation, at all nephrology centers, of an Advanced Renal Disease Clinic (MaReA). The nephrologist coordinates a health professional team (nurses, psychologists, dieticians) and establishes the timing, means of access and check-ups, and plans the replacement therapy implementation process. Data from treated patients is registered in a regional database, connected to the Dialysis and Transplant Register. After 4 years, many positive aspects have been highlighted as well as several issues. Greater attention was paid to the various aspects related to initiation of replacement therapy—a more precocious, sometimes pre-emptive, entry onto the transplant list has been achieved, as well as renewed interest in home treatment (peritoneal dialysis and hemodialysis); collaboration with dietetic services has been activated. The latter services have proven problematic in some situations, due in part to a simultaneous regional health reorganization. One solution will be sought in a closer cooperation with the regional network of clinical nutrition.

To better manage CKD progression and rationalize interventions in all their aspects, it will be necessary to extend the scope of action to include earlier stages of renal damage and involve those operating in the local community (general practitioners, health districts). It is necessary to seize the opportunity offered by the National Chronicity Plan [143], which Italian regions must adopt, to develop processes and pathways which provide nephrologists with the appropriate tools to coordinate clinical activity needed for a  proper CKD patient care.

## Dietetic nutritional support levels: full/part-time dietitian dedicated to nephrology, in-hospital dietitian, informative materials, digital supports

The dietitian involved in CKD nutritional treatment participates, in collaboration with the nephrologist, in the dietetic-nutritional program through evaluation of the patient’s nutritional status and development of a personalized dietary plan. In addition, the dietician interacts with other team members (psychologists, physiotherapists, etc.) to identify and promote individual factors that may favor adherence to the overall therapeutic plan [144]. A dietitian-provided DNT is recommended for CKD patients from stage 1 up to stage 5, including dialysis and transplant [145]. The dietician, in collaboration with the nephrologist, involves the patient and the caregiver in all stages of nutritional treatment, from dietary history to formulation, construction and implementation of the dietary plan. The dietitian’s role is to provide adequate information and education, aimed at self-management of the diet in accordance with specific shared objectives.

The National (Italian) Dietitians Association (ANDID) supports the recommendation from the National Kidney Foundation (NKF) that an expert dietitian should be available for every 150 nephropathic patients [84]. Visit length is a factor that affects the quality of DNT [144]. Available evidence shows that 60–90 min are needed for the first appointment and 45–60 min for subsequent visits [146]. The European Guidelines for Nutritional Assistance of adult patients with CKD emphasize that a protein intake < 0.8 g/kg of ideal body weight (IBW)/day is not to be prescribed if a renal dietitian is not available [147].

The hospital dietician, full-time or part-time dedicated to nephrology, carries out his/her activity in collaboration with the other professionals involved in the assistance, adapting the DNT to the different stages of CKD. He/she carries out pedagogical-educational and informational activities, aimed respectively at health care and the collective catering personnel. The dietician develops dietetic tables for the different stages of CKD, making specifications for purchase of very low protein dietary products and menus for patients of any age and background. The hospital dietician organizes and coordinates the various people involved in feeding services in order to ensure compliance with low-protein diet protocols. He/she ensures continuity of care through development of a personalized diet plan to be implemented after discharge.

Ability to correctly combine biological and psycho-social aspects of dietary history is a central skill of the dietician expert in nutritional treatment of CKD. This ability allows for development of personalized diet plans that meet both the patient’s tastes and needs [148]. In the case of elderly patients, paper supports are to be illustrated with involvement of family members or caregivers in order to guarantee the best possible practical application. Exchange lists, informative brochures, and indications based on traditional eating habits are also elaborated with team collaboration and must guarantee patients a wide variety of choices, limiting monotony and the feeling of deprivation associated with traditional approaches [149]; emphasis must be placed on what can and should be consumed rather than on what is “forbidden”. A telephone consultancy service/web platform could be a support in the situations where a dietician is not available. Establishment of this service should include prescription of a therapeutic plan by the nephrologist, development of a DNT plan by the dietiian, and subsequent validation by the nephrologist treating the patient. Various attempts have been made to produce computer programs that create diet plans, but these do not guarantee protocol customization and limit the safety and effectiveness.

## Adherence to a dietary prescription is critical, as with pharmacological therapies. Proper information and education when implementing a diet program remain at the basis of appropriate chronic patient management

In the guideline document for CKD, patient dissatisfaction about being poorly informed and involved in their own care emerges [150]. The decree on chronicity has patient management at its core. Much remains to be done, therefore, in order to involve renal patients in treatment adherence, which is essential for improving results and reducing healthcare costs [151–153]. The term “compliance” defines the degree to which patient awareness coincides with the recommendations provided by health professionals [80] and it is often used as a synonym for “adherence”. In general, patient adherence to dietetic nutritional therapy is about 31%. Patient involvement is a systematic pathway that identifies and characterizes possible means of interaction among the patient, family, caregiver, and the health workers. Involvement is a function of the gradual choice of patients to take an active role in their health management. This process is influenced by individual, social, environmental and socio-economic factors.

It is very difficult to act upon processes related to the disease of a single patient. It is easier to act upon the patient care team’s processes of awareness, information, management and preparation. Patient and patient care team involvement must become the rule within clinical practice and be measured with appropriate validated scales [154], as they are part of the national evaluation and accreditation criteria, and Joint Commission International (JCI) certification, at the points referring to aspects of patient communication and support for self-management. Fundamental to the success of any type of treatment is management [155, 156] that can only take place if there is a correct involvement of the patient, family, caregivers and health professionals (empowerment). Training is essential for the development of skills [157] and must be assessed and measured if a real desire to increase the effectiveness and efficiency of clinical-care interventions is present. Acquisition of communication techniques is fundamental and of support for better patient and family member information and involvement. Audiovisual systems, applications, and social networks favor the engagement process [158]. Videos can be powerful tools as sources of narrative medicine, capturing more easily the patient attention.

## Healthcare professionals involved in patient management with CKD 4–5 should promote regular physical activity as an integral part of dietetic-nutritional therapy

Physical activity is a key element in chronic disease prevention, as it improves many aspects including blood pressure control, glucose and lipid metabolism, nutritional status, and endothelial function [159]. In contrast, the literature is consistent in indicating how sedentariness is associated with an increased risk of morbidity and mortality [160–162]. There are now strong data supporting the favorable effects of physical activity in patients with CKD who are on a low protein  diet [163], including elderly patients [164]. In these patients, physical activity can represent an important anabolic stimulus that favors nutrient use and contrasts loss of lean muscle [163]. Despite this evidence, physical activity is rarely prescribed to CKD patients [165].

One of the nephrologist’s tasks is to overcome the obstacles that frequently oppose implementation of “rehabilitative” programs [166]. Lack of knowledge and awareness of the importance of physical activity in CKD demonstrates the need to create multidisciplinary teams in order to implement appropriate physical activity programs for renal patients [167]. Healthcare professionals involved in CKD patient management should promote regular physical activity as an integral part of DNT, especially in advanced stages of the disease.

## Conclusion

The following 20 consensus statements have been defined to underline several relevant aspects of the nutritional approach in patients with advanced chronic renal insufficiency. These statements were prepared, discussed, and shared by Italian nephrologists, dietiians, and patients. We believe that they may promote a successful and safe implementation of nutritional treatment of CKD patients.

1. In patients with CKD 4–5, a diet with non-monitored intake of calories, protein, sodium, and phosphates hastens and exacerbates clinical metabolic alterations related to advanced CKD.
2. In patients with CKD 4–5, a diet with non-monitored intake of energy, protein, sodium, and phosphate can reduce the effectiveness of drug therapy or require an increase of dose.  
3. Lack of metabolic compensation, with the appearance of uremic signs and symptoms, is an indication to begin dialysis treatment, in the same way as and independently of the level of residual renal function.
4. Untreated chronic renal insufficiency leads to undernourishment due to loss of appetite, nausea, and anorexia.
5. Considering the pathophysiology of advanced chronic renal insufficiency, an adequate dietetic-nutritional therapy provides: reduction of protein intake, reduction of phosphorus intake, reduction/monitoring of sodium intake, monitoring of potassium intake, limitation of the fixed acid load.
6. To ensure the effectiveness of dietetic-nutritional therapy in chronic renal insufficiency, compliance with the following conditions must be verified: satisfaction of the caloric needs, adequate intake of essential amino acids, correction of metabolic acidosis, good glyco-metabolic control
7. Protein-free products, made up of carbohydrates, contain almost no protein, phosphorus, sodium, and potassium. They raise caloric intake while leaving more space for foods rich in high biological value proteins which guarantee essential amino acid intake. Better therapeutic efficacy is thus obtained with a lower risk of nutritional inadequacy.
8. Essential amino acid and keto acid mixture  is an useful supplementation in CKD patients; it is mandatory on a very low protein diet.
9. DNT in CKD 4–5 must be managed according to the stages and criteria of any other drug therapy: indications, contraindications, side effects, changes in dosage, verification of results, follow-up.
10. Regular evaluation of the nutritional and functional status of patients with CKD 4–5 at the start of treatment and during follow up is essential for diet management.
11. Proper low protein nutritional therapy does not cause malnutrition in the short or long term.
12. Proper dietetic-nutritional therapy in advanced CKD may delay the need for replacement therapy. For this reason, its use is especially indicated for patients in the preemptive kidney transplant programme.
13. In selected patients, a proper dietetic-nutritional therapy can allow an integrated care approach with once-a-week hemodialysis schedule.
14. Appropriate DNT can allow an integrated incremental peritoneal dialysis regime.
15. Advanced CKD is characterized by a dysbiosis of the intestinal microbiota, which contributes to uremic intoxication and cardiovascular damage. A hypoproteic nutritional therapy, associated with adequate fiber intake, can counteract dysbiosis and reduce the production of uremic toxins.
16. In terms of pharmacoeconomics, an appropriate Dietetic Nutritional Therapy allows for cost and resource savings in the management of patients with advanced chronic renal failure.
17. For a more effective and easier clinical management of advanced CKD, it is necessary to implement organizational models integrating various health professionals.
18. Dietetic nutritional support levels: full/part-time dietitian dedicated to Nephrology, in-hospital dietitian, informative materials, digital supports
19. Adherence to a dietary prescription is critical, as with pharmacological therapies. Proper information and education when implementing a diet program remain at the basis of appropriate chronic patient management.
20. Healthcare professionals involved in patient management with CKD 4–5 should promote regular physical activity as an integral part of the dietetic-nutritional plan.

## Abbreviations

BMI: Body mass index
CDDP: Combined diet dialysis program
CKD: Chronic kidney disease
DASH: Dietary approaches to stop hypertension
DNT: Dietetic-nutritional therapy
EAA: Essential amino acid
ESA: Erythropoiesis-stimulating agents
GFR: Glomerular filtration rate
IBW: Ideal body weight
ICP: Integrated care pathways
IDDP: Integrated diet dialysis program
IPD: Incremental peritoneal dialysis
KA: Keto-acids
MIS: Malnutrition inflammation score
NNT: Numbers needed to treat
PD: Peritoneal dialysis
PEW: Protein-energy wasting
PTH: Parathormone
pmp: Patients per million population
QALY: Quality-adjusted life-year
RAAS: Renin–angiotensin–aldosterone system
RAPA: Rapid assessment of physical activity
RRF: Residual renal function
SCFA: Short-chain fatty acids
SGA: Subjective global assessment
sVLPD: Supplemented very low protein diet
VLPD: Very low-protein diet

## Footnotes

1 Demonstrated in healthy subjects/ clinical contexts different from  CKD.
